# Experimental evidence-based construction of electroacupuncture for ischemic stroke: a meta-analysis and systematic review

**DOI:** 10.3389/fneur.2025.1491132

**Published:** 2025-02-05

**Authors:** Yaoguang Guo, Sihan Hu, Shiman Luo, Lang Tu, Yao Tang, Fang Zeng

**Affiliations:** ^1^Department of Acupuncture and Moxibustion, Hospital of Chengdu University of Traditional Chinese Medicine, Chengdu, China; ^2^Department of Integrated Traditional Chinese and Western Medicine, Peking University First Hospital, Beijing, China; ^3^Institute of Integrated Traditional Chinese and Western Medicine, Peking University, Beijing, China; ^4^State Key Laboratory of Southwestern Chinese Medicine Resources, School of Pharmacy, Chengdu University of Traditional Chinese Medicine, Chengdu, China; ^5^West China School of Public Health and West China Fourth Hospital, Sichuan University, Chengdu, Sichuan, China; ^6^Acupuncture and Tuina School, Chengdu University of Traditional Chinese Medicine, Chengdu, China; ^7^Acupuncture and Brain Science Research Center, Chengdu University of Traditional Chinese Medicine, Chengdu, China; ^8^Key Laboratory of Acupuncture for Senile Disease (Chengdu University of TCM), Ministry of Education, Chengdu, China

**Keywords:** electroacupuncture, ischemic stroke, meta-analysis, systematic review, review

## Abstract

**Objective:**

Ischemic stroke represents a leading cause of disability and mortality worldwide, necessitating effective and complementary therapeutic strategies. Electroacupuncture (EA), a modern extension of traditional acupuncture, has garnered attention for its potential neuroprotective effects in ischemic stroke rehabilitation. This meta-analysis and systematic review aim to synthesize current experimental evidence on the efficacy of EA in ischemic stroke models, focusing on neurological outcomes, infarct volumes, and underlying molecular mechanisms.

**Methods:**

A comprehensive search was performed across four databases—Cochrane Library, EMBASE, PubMed, and Web of Science—to identify relevant experimental studies that utilized electroacupuncture (EA) as a therapeutic modality for ischemic stroke in animal models. This search encompassed all literature available from the inception of each library through December 2023. Studies were rigorously screened based on predefined inclusion and exclusion criteria. Data on cerebral infarction volume, neurological deficit scores, cellular apoptosis, and molecular pathways were extracted and analyzed.

**Results:**

Eleven eligible studies involving 302 animals (151 in EA treatment groups and 151 in control groups) were included. Meta-analysis revealed that EA significantly reduced cerebral infarction volumes [MD = −15.78, 95%CI (−21.40, −10.16), *p* < 0.05] and TUNEL-positive cells [MD = −26.46, 95%CI (−40.40, −12.51), *p* < 0.05], indicating reduced apoptosis. Improvements were also noted in neurological deficit scores [MD = −0.59, 95%CI (−0.92, −0.27), *p* < 0.05] and modified Neurological Severity Scores (mNSS) [MD = -5.68, 95%CI (−7.41, −3.95), *p* < 0.05], highlighting functional recovery. While the analysis showed no significant effect on caspase-3 densities [MD = −0.39, 95%CI (−0.79, 0.02), *p* > 0.05], a notable increase in Bcl-2 densities suggested an anti-apoptotic mechanism [MD = −0.73, 95%CI (−1.68, 0.21), *p* > 0.05]. The heterogeneity of the included studies points to complex underlying mechanisms, potentially involving modulation of apoptotic pathways and cerebral blood flow.

**Conclusion:**

This meta-analysis substantiates the neuroprotective potential of EA in ischemic stroke models, primarily through apoptosis modulation and possibly through improved cerebral perfusion. These findings advocate for the integration of EA into stroke rehabilitation protocols and underscore the need for clinical trials to validate its efficacy in human subjects. Our study not only reinforces the therapeutic value of EA but also prompts further investigation into its underlying mechanisms, potentially guiding more effective stroke recovery strategies.

## 1 Introduction

Ischemic stroke, characterized by the abrupt loss of cerebral blood flow due to an occlusion, precipitates a cascade of pathophysiological events culminating in extensive neuronal damage (1–3). The complexity of these events, encompassing excitotoxicity, oxidative stress, inflammation, and apoptosis, presents a formidable challenge for therapeutic intervention (4–8). Traditional pharmacological treatments, while invaluable, often offer limited windows of efficacy and are frequently accompanied by substantial side effects. In this context, electroacupuncture (EA) presents a unique multimodal approach, purportedly targeting multiple pathways implicated in ischemic injury.

Electroacupuncture, a modern adaptation of the ancient Chinese acupuncture technique, involves the application of electrical stimulation to traditional acupuncture points (9, 10). Its potential neuroprotective effects, mediated through various biological and physiological mechanisms, offer a promising avenue for post-stroke rehabilitation (11, 12). Preliminary investigations into EA’s efficacy have reported promising outcomes, including improved cerebral perfusion, reduced infarct volume, and enhanced neurological function (13–15). These effects are hypothesized to stem from EA’s ability to modulate neurovascular and pathways, promote neurogenesis, and inhibit (16–20). However, the diversity of experimental designs, acupuncture protocols, and outcome measures across studies necessitates a comprehensive analysis to distill these findings into actionable clinical insights.

This research seeks to bridge this gap through a systematic review and meta-analysis of the existing literature on EA’s effects on ischemic stroke models. By aggregating data from diverse studies, this work aims to quantify EA’s impact on key outcomes such as cerebral infarction volume, neuronal apoptosis rates, and functional recovery scores. Furthermore, this study will employ advanced statistical techniques to explore the heterogeneity among findings and assess the robustness of the evidence. In doing so, it will not only provide a consolidated overview of EA’s efficacy but also identify knowledge gaps and directions for future research.

In addressing these objectives, this study will contribute to the field by offering a rigorous, evidence-based assessment of EA’s role in ischemic stroke rehabilitation. It will also propose a conceptual framework for understanding the complex interplay of biological processes involved in EA-mediated neuroprotection, thereby paving the way for targeted clinical applications and further scientific inquiry.

## 2 Methods and materials

The methods used in this study followed the meta-analysis criteria in the PRISMA guidelines. The meta-analysis did not need to follow ethical review and was also approved by Chengdu University of Traditional Chinese Medicine.

### 2.1 Retrieval strategy

We conducted comprehensive searches across four major databases: PubMed, Web of Science, Embase, and the Cochrane Library, covering the period from their inception up to December 31st, 2023. Our search strategy was specifically formulated. The search method is: “electroacupuncture” and “ischemic stroke,” to ensure the retrieval of relevant studies pertaining to the efficacy of electroacupuncture in the treatment of ischemic stroke.

### 2.2 Eligibility criteria

The inclusion criteria were pre-specified as follows: (1) Established experimental animal models of ischemic stroke; (2) The intervention group received electroacupuncture treatment; (3) The model group received sham acupuncture intervention or no treatment; (4) There were no restrictions on animal species, gender, age, weight, and sample size; (5) The primary outcome measures included neurological function scores, infarct size or ratio, and all markers indicative of ischemic stroke.

The exclusion criteria for the documents were rigorously defined as follows: (1) Exclusion of reviews, *in vitro* studies, and trials; (2) Studies not involving electroacupuncture for prevention; (3) Research focusing on electroacupuncture in the treatment of diseases other than ischemic stroke; (4) Elimination of duplicate publications; (5) Exclusion of unpublished literature.

### 2.3 Data extraction

The data extraction table was independently completed by two authors to ensure accuracy. Additionally, the literature table includes the following information: (1) the first author’s name and the year of study publication; (2) detailed information about the animal model, such as species, sex, and weight; (3) the induction method of ischemic stroke in the animal model; (4) detailed information of the intervention group, including selected acupoints and details of electroacupuncture stimulation; (5) information on sham acupuncture if used in the model group; (6) the main conclusions of the study; (7) outcome indicators. If experimental animals received varying stimulation intensities or intervention durations, only the data from the highest intensity or longest duration were taken. If data were presented in graph form, they were measured using digital ruler software. We attempted to obtain further information by contacting the authors when primary data were incomplete.

### 2.4 Risk of bias

To assess the quality of literature on electroacupuncture treatment in ischemic stroke animal studies, the CAMARADES checklist with 10 criteria proposed by Macleod was employed. The assessment included: (1) Sample size calculation; (2) Random sequence generation; (3) Blinded induction; (4) Blinded outcome assessment; (5) Use of anesthetics without significant neuroprotective activity; (6) Appropriateness of animal models; (7) Temperature control statement; (8) Publication in peer-reviewed journals; (9) Compliance with animal welfare laws; (10) Declaration of potential conflicts of interest. Each study was scored on a scale of 10, with one point for each criterion. The Risk of Bias Assessment was independently conducted by two researchers, involving a third in cases of discrepancy.

### 2.5 Quantitative synthesis and other statistical analyses

For the meta-analysis of animal studies, a quantitative synthesis was adopted. Outcomes, quantified as standardized mean differences, were accompanied by 95% confidence intervals. Statistical significance was determined at *p* < 0.05 for contrasts between experimental and control groups. Heterogeneity assessment employed the I-square statistic, with *I^2^* values above 50% denoting considerable heterogeneity. Given the investigative nature of these studies, random effects models were uniformly utilized, independent of the *I^2^* value. In addition, we performed a sensitivity analysis by sequentially excluding individual studies to assess the robustness and stability of the results of the meta-analysis.

## 3 Results

### 3.1 Research screening

**Figure 1:**
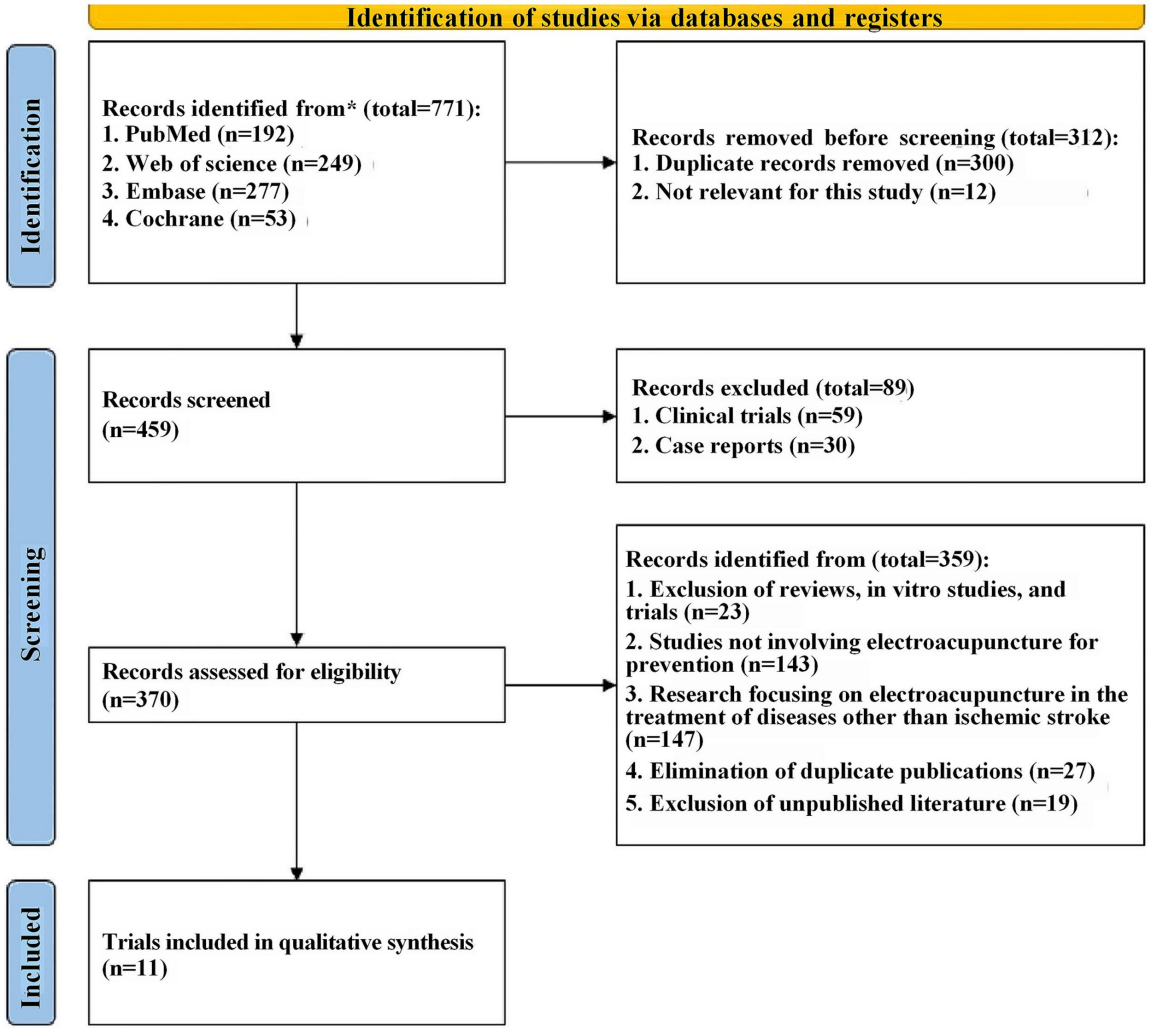
PRISMA-type flow diagram.

Firstly, a total of 771 relevant records were retrieved from four databases through a detailed search strategy. The records were then imported into Endnote X9 and 312 records were excluded due to duplication and other reasons. Upon title and abstract screening, 89 were excluded for not meeting inclusion criteria, leaving 370 full-text articles for detailed review, subsequently, 359 articles were excluded for reasons including *in vitro* experiments, no use of electroacupuncture therapy, etc. Finally, a total of 11 eligible English studies were included in this meta-analysis (13, 15, 21–29) (The detailed document scheduling process is shown in Figure 1).

### 3.2 Features of included researches

The 11 articles included a total of 302 animals, of which the experimental group (n = 151) was receiving EA treatment, and the control group (*n* = 151) was treated with sham EA. All experiments were carried out on male rodents, predominantly Sprague–Dawley rats (96.03%) and the rest were C57BL/6 mice. The rats weighed between 150 and 320 g, while the mice weighed between 16 and 18 g. Regarding the use of anesthetic in experimental animals, chloral hydrate is used in 72.73% (8/11) articles, and isoflurane is used in 27.27% (3/11) articles.

In terms of electroacupuncture treatment, 81.82% (9/11) of the articles used electroacupuncture for 30 min each time, whereas two articles EA treatments for durations of both 15 and 20 min, respectively (Details are in Table 1).

**Table 1:** List of key characteristics in all 11 studies based on full-text assessment.

Author(s)/Year	Species and strains	Gender (M/F)	Sample size EA/NEA	Weight of the animal	Model approach	Criteria of successful model establishment	Anesthetic	EA (wave, intensity, frequency, time)	NEA group	Outcome parameters
Bin Chen 2021	Sprague–Dawley (SD) rats	Adult Male	26/26	280–300 g	MCAO	Ischemic rats that showed a sharp decrease to 20% of baseline (before ischemia) were used for further experimentation	1.5% isoflurane	sparse-dense wave, intensity of 1 mA, frequency of 4/20 Hz, 30 min	Only Anesthetic	(1) TTC, (2) Neurological deficit score 1–3, (3) cleaved caspase-3(%), (4) NeuN (%), (5) Histological deficit score, (6) Density of TUNEL+ cell (per mm2), (7) Density of NeuN+cell (per mm2), (8) Density of cofilin rod (per mm2), (9) Relative intensity of MAP2 (%)
Laiting Chi 2018	Sprague–Dawley (SD) rats	Adult Male	NM	220–250 g	MCAO	The rats showed a sharp reduction in rCBF (The regional cerebral blood flow) of at least 70%	Isoflurane (2% induction and 1.5% maintenance)	an intensity of 1 mA and a 2/15 Hz sparse-dense frequency for 30 min	on the ulna side of the metacarpus served	(1) TTC, (2) TNF-a level in peri-infarct area, (3) IL-10 level in peri-infarct area, (4) Serum TNF-a level, (5) Serum IL-10 level，(6) MDA level, (7) SOD activity, (8) muscarinic receptors M1—5, (9) 7nAChR
Xin-yin Xu 2020	Sprague–Dawley (SD) rats	Male	24/24	200–220 g	MCAO	mNSS scores of 7 to 12 were regarded as successful models	10% chloral hydrate	Intensity (1 -2 mA) 2 Hz, 0.2 ms pulse, 15 min	No intervention	(1) TTC, (2) IL-1β, (3) IL-6, (4) IL-8, (5) Bcl2 positive cells counted, (6) Bax positive cells counted, (7) Caspase-3 positive cells counted
Xing Ying 2018	Sprague–Dawley (SD) rats	Male	18/18	280–320 g	MCAO	Rats with score 1–3 points demonstrated successful establishment of MCAO model	10% chloral hydrate	dense-disperse waves of 4/20 Hz (adjusted to the muscle twitch threshold), intensity of 1 mA, and peak voltage of 6 V	No intervention	(1) TTC, (2) Neurological deficit score 1–3, (3) caspase-3 activity, (4) Bcl-2 activity, (5) Bim activity, (6) caspase-3 relative densities, (7) Bcl-2 relative densities, (8) p-ERK1/2 relative densities, (9) p-SAPK/JNK relative densities, (10) p-p38 relative densities, (11) The expression of MK, (12) Immunohistochemical staining of MK
Zhou Heng 2017	Sprague–Dawley (SD) rats	Male	NM	250–280 g	MCAO	NM	1. 10% chloral hydrate, 2. 2, 3, 4% isoflurane anesthesia	an intensity of 1 mA and dilatational wave	No intervention	(1) TTC, (2) the neurological scores (the method of Garcia), (3) No. of TUNEL(+) Cells (mm^2^), (4) miR-132-5p in the penumbra cortex, (5) miR-191a-5p in the penumbra cortex, (6) miR-212-5p in the penumbra cortex, (7) miR-223-5p in the penumbra cortex, (8) miR-1298
Xing Ying 2018	Sprague–Dawley (SD) rats	Male	24/24	280–320 g	MCAO	Doppler flowmetry	10% chloral hydrate	disperse-dense waves of 4 or 20 Hz, output voltage of 4 V for 30 min	No intervention	(1) TTC, (2) Neurological deficit score 1–3, (3) TUNEL-positive cells (100%), (4) caspase-3 relative densities, (5) Bcl-2 relative densities, (6) the ratios of caspase-3 positive cell, (7) cleaved caspase-3, (8) Bim, (9) p-PTEN, (10) p-PDK1, (11) p-Akt (Thr308), (12) P-GSK-3β, (13) p-Akt (Ser473), (14) t-Akt
Ruisi Tian 2019	C57 mice	Male	6/6	16–18 g	MCAO	Neurological deficits	4% chloral hydrate	an intensity of 1 mA and a frequency of 2/15 Hz for 20 min	No intervention	(1) TTC, (2) TNF-α, (3) No. of TUNEL(+) Cells, (4) apoptotic index(%) TUNEL-positive cells (100%), (5) Bcl-2 activity, (6) Bax protein Expression level, (7) the ratios of caspase-3 positive cell, (8) Notch3, (9) Jagged1, (10) HES1
Siqiao Cao 2021	Sprague–Dawley (SD) rats	Male	15/15	250 ± 30 g	MCAO	NM	10% chloral hydrate	waveform density wave, frequency 4/20 Hz, current 1_3 mA, voltage 1_3 V, timing 30 min	No intervention	(1) TTC, (2) No. of TUNEL(+) Cells (mm^2^), (3) apoptotic index(%) TUNEL-positive cells (100%), (4) miR-34, (5) miR-235, (6) miR-275, (7) LC3II /LC3I ratio, (8) LC3 positive cells(%), (9) P62,10
Ranran Ma 2016	Sprague–Dawley (SD) rats	Male	50/50	280–320 g	MCAO	NM	10% chloral hydrate	An intensity of 1 mA and frequency of 2 Hz s for 30 min	No intervention	(1) TTC, (2) mNSS 0–18, (3) apoptotic index (%) TUNEL-positive cells (100%), (4) Bcl-2 relative densities, (5) Bax protein Expression level, (6) The number of BrdU+ cells, (7) MMP-9, (8) TIMP-1,9
Xiehua Xue 2014	Sprague–Dawley (SD) rats	Male	12/12	280–320 g	MCAO	NM	10% chloral hydrate	disperse wave of 4 and 20 Hz for 30 min	No intervention	(1) TTC, (2) Neurological deficit score 1–3, (3) apoptotic index(%) TUNEL-positive cells (100%), (4) Western blot Bcl-2/Bax ratio (% of SC), (5) cleaved caspase-3 positive rate (%), (6) p-Akt/t-Akt ratio, (7) p-Bad/Bad,8
Yu Ri Kim 2013	Sprague–Dawley (SD) rats	Male	6/6	150–180 g	MCAO	laser-Doppler flowmeter	2.0% isoflurane	2 Hz stimulation for 30 min and intensity was set at 1 mA	No intervention	(1) TTC, (2) No. of TUNEL(+) Cells (mm2), (3) caspase-8 activity (% of sham), (4) caspase-9 activity (% of sham), (5) caspase-3 activity (% of sham)

### 3.3 Quality evaluation

**Figure 2:**
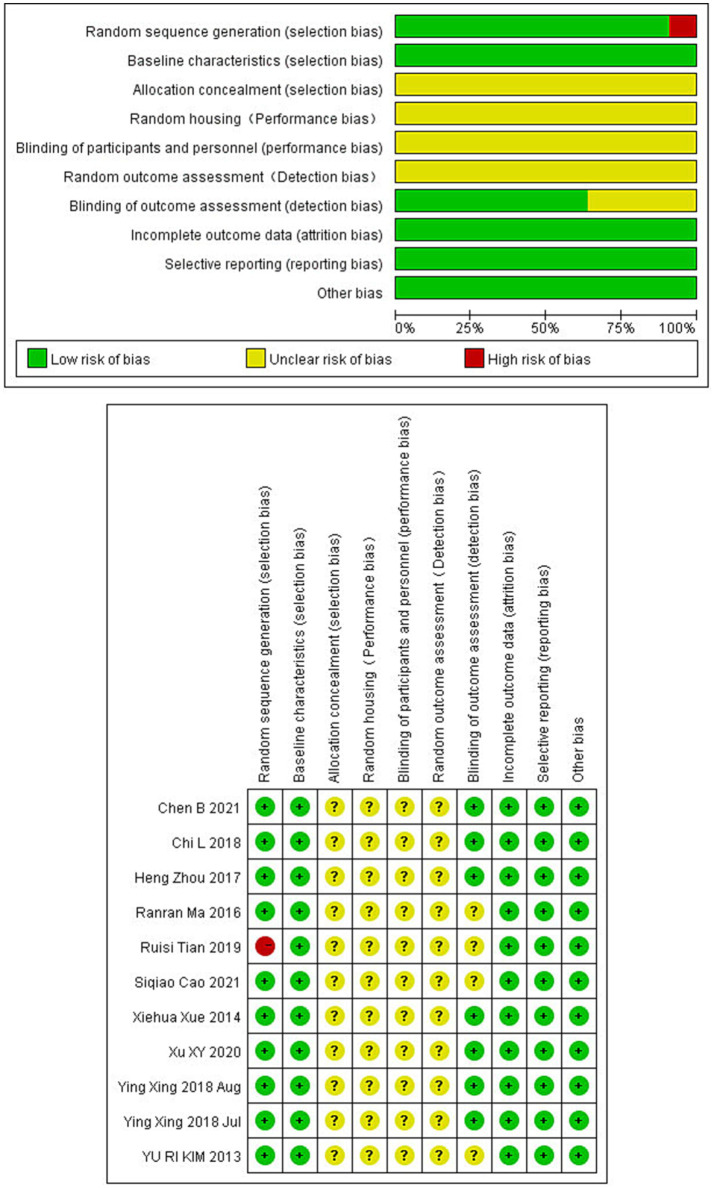
Bias risk in studies on electroacupuncture for ischemic stroke treatment.

The rigor of our selected studies was upheld by consistent baseline characteristics and the absence of incomplete data, minimizing reporting bias. However, the lack of explicit blinding of outcome assessors in four studies introduces a potential limitation. Other aspects such as allocation concealment, randomization, and blinding procedures varied, introducing elements of uncertainty in bias evaluation. All the details are shown in Figure 2.

### 3.4 Pathological analysis

The pathological results can intuitively demonstrate the therapeutic effect of EA on ischemic stroke animals, so the pathological results of the included studies were qualitatively analyzed. Triphenyl tetrazolium chloride (TTC) staining was used in all the 11 included articles, and the results showed that the infarct size of the EA group was smaller than that in the control group, and the difference was statistically significant. Notably, EA treatment exhibited a robust neuroprotective effect, particularly by reducing middle brain region damage. Hematoxylin–eosin (H&E) staining (*n* = 1) revealed that EA treatment could effectively improve neuronal necrosis and pathological tissue disorder in cerebral infarction tissue. Immunohistochemical staining (*n* = 8) indicated that ischemic stroke could significantly increase the number of caspase-3 positive cells, while EA treatment could reduce the expression of apoptosis-related proteins, such as caspase-3, and it also inhibit the expression of a7nAChR mRNA, preventing the damage of central cholinergic system. Additionally, the number of BrdU+ /GFAP+(red) cells were significantly decreased by EA treatment. One article found that there was autophagosome accumulation in the electroacupuncture treatment group by transmission electron microscopy. However, compared with the control group, the electron density of the autophagy structure was lower and the autophagy structure was different.

### 3.5 Neuroprotection and brain injury reduction

#### 3.5.1 Cerebral infarction volume

**Figure 3:**
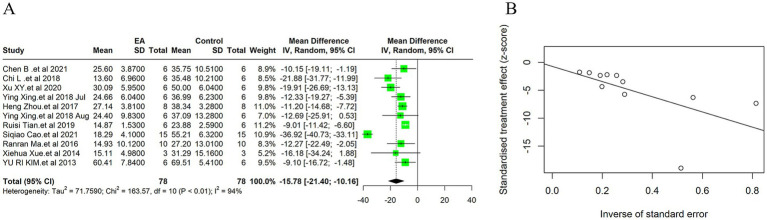
Meta-analytic assessment of electroacupuncture’s efficacy in reducing cerebral infarction volume. **(A)** The forest plot. **(B)** Egger’s test funnel plot.

Due to the high heterogeneity among the 11 included studies (*I^2^* = 94%, *p* < 0.01), a random effect model was used for data analysis, and the meta-analysis revealed statistically significant result: Compared with the control group, EA treatment could significantly reduce the cerebral infarction volume in the ischemic stroke animals [MD = −15.78, 95%CI (−21.40, −10.16), *p* < 0.05] (Figure 3A). This substantial decrease underscores EA’s neuroprotective efficacy, aligning with the mechanistic insights suggesting EA’s role in modulating inflammatory responses and enhancing cerebral blood flow. Considering the subjectivity of the funnel plot (Supplementary Figure 2A), Egger’s test was used to explore publication bias, and the result was (*t* = −0.30, *p* = 0.77 > 0.05) indicates no publication bias (Figure 3B), indicating that it had little impact on our results. In the sensitivity analysis, we calculated the 95% confidence intervals of the relevant parameters. According to the results of the analysis, the 95% confidence intervals for the relevant variables are [−2.46, −1.60] (Supplementary Figure 3).

### 3.6 Apoptosis indicators

#### 3.6.1 TUNEL positive cells

**Figure 4:**
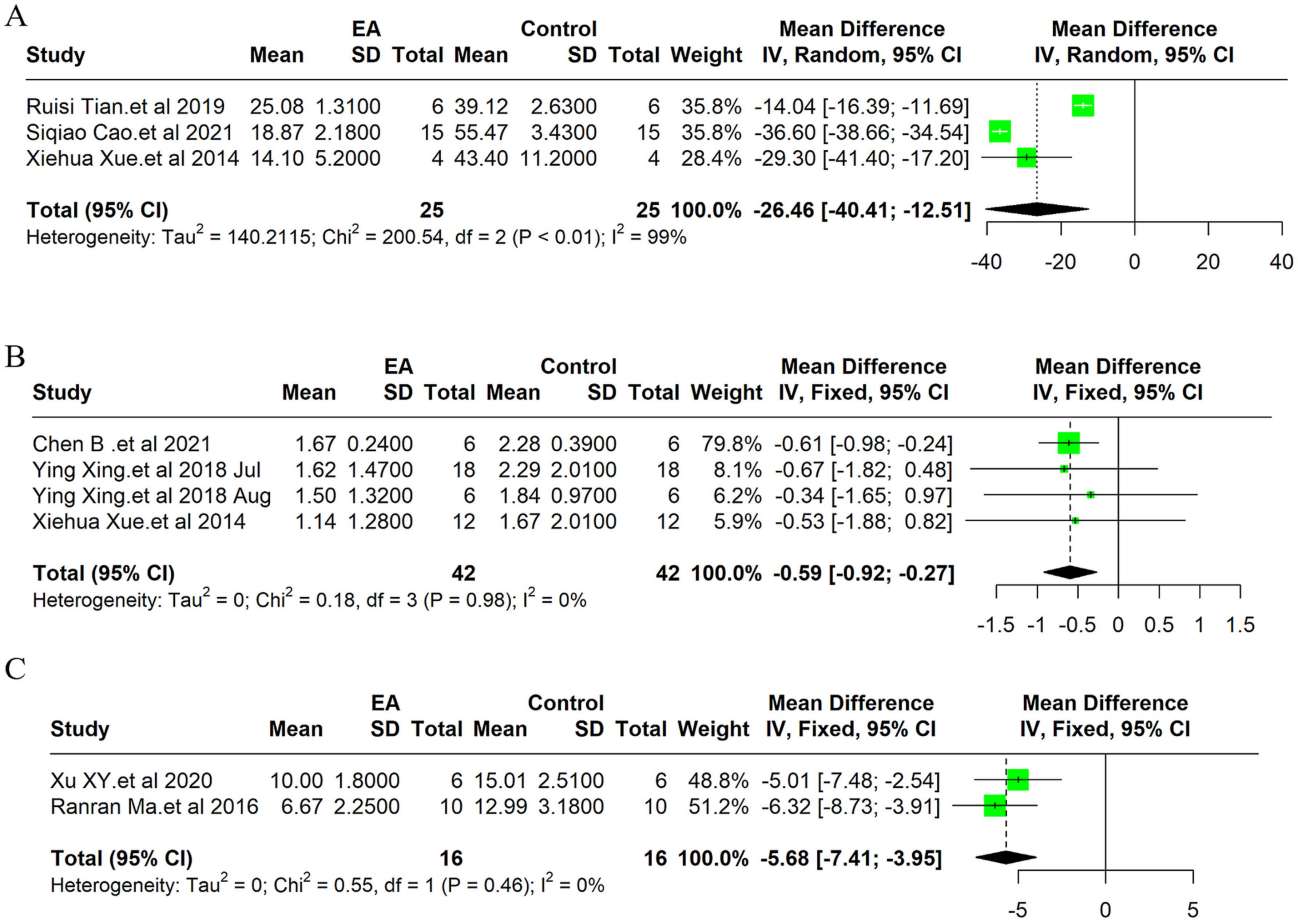
Meta-analysis of electroacupuncture’s efficacy in ischemic stroke models: forest plots of **(A)** TUNEL positive cells, **(B)** Neurological deficit scores **(C)** the modified neurological severity scores (mNSS).

Due to the high heterogeneity of the 3 included studies (*I^2^* = 99%, *p* < 0.01), a random effect model was used for data analysis, and the meta-analysis revealed statistically significant result: Compared with the control group, EA treatment could significantly reduce the TUNEL positive cells in the ischemic stroke animals [MD = −26.46, 95%CI (−40.40, −12.51), *p* < 0.05] (Figure 4A). Indicates EA’s role in reducing cell death by apoptosis, a key aspect of neuroprotection. The funnel plot was made to explore publication bias, and the results showed that there was a slight publication bias (Supplementary Figure 2B). EA’s influence on cellular apoptosis was evident, with a significant reduction in TUNEL positive cells in treated subjects, further validating EA’s neuroprotective role.

#### 3.6.2 Caspase-3 relative densities

Due to the high heterogeneity of the 2 included studies (*I^2^* = 76%, *p* < 0.05), a random effect model was used for data analysis, and the meta-analysis revealed this result: there was no statistically significant difference in the caspase-3 relative densities between the EA treatment group and control group, EA treatment could not reduce the caspase-3 relative densities in the animals with ischemic stroke [MD = −0.39, 95%CI (−0.79, 0.02), *p* > 0.05] (Supplementary Figure 1A). This suggests that while EA exhibits neuroprotective properties, its mechanism may not primarily involve the modulation of caspase-3 mediated apoptotic pathways. The funnel plot was made to explore publication bias, and the results showed that there was a slight publication bias (Supplementary Figure 2C).

#### 3.6.3 Bax protein expression level

Due to the high heterogeneity of the 2 included studies (*I^2^* = 99%, *p* < 0.01), a random effect model was used for data analysis, and the meta-analysis revealed this result: there was no statistically significant difference in the Bax protein expression level between the EA treatment group and control group, EA treatment could not reduce the Bax protein expression level in the animals with ischemic stroke [MD = −0.73, 95%CI (−1.68, 0.21), *p* > 0.05] (Supplementary Figure 1B). The funnel plot was made to explore publication bias, and the results showed that there was a significant publication bias (Supplementary Figure 2E).

#### 3.6.4 Apoptotic index

Due to the high heterogeneity of the 2 included studies (*I^2^* = 67%, *p* < 0.05), a random effect model was used for data analysis, and the meta-analysis revealed statistically significant result: Compared with the control group, EA treatment could significantly reduce the apoptotic index in the ischemic stroke animals [MD = −22.14, 95%CI (−34.72, −9.56), *p* < 0.05] (Supplementary Figure 1C). The funnel plot was made to explore publication bias, and the results showed that there was a slight publication bias (Supplementary Figure 2F).

### 3.7 Neurological scores

#### 3.7.1 Neurological deficit score

Due to the low heterogeneity of the 4 included studies (*I^2^* = 0%, *p* > 0.05), a fixed effect model was used for data analysis, and the meta-analysis revealed statistically significant result: Compared with the control group, EA treatment could significantly reduce the neurological deficit score in the ischemic stroke animals [MD = −0.59, 95%CI (−0.92, −0.27), *p* < 0.05] (Figure 4B). This improvement underscores EA’s efficacy not only in reducing cellular and molecular markers of ischemic damage but also in enhancing functional recovery post-stroke. The funnel plot was made to explore publication bias, and the results showed that there was a slight publication bias (Supplementary Figure 2G).

#### 3.7.2 Neurological function score of mNSS

Due to the low heterogeneity of the 2 included studies (*I^2^* = 0%, *p* > 0.05), a fixed effect model was used for data analysis, and the meta-analysis revealed statistically significant result: Compared with the control group, EA treatment could significantly reduce the modified Neurological Severity Scores (mNSS) scores in the ischemic stroke animals [MD = −5.68, 95%CI (−7.41, −3.95), *p* < 0.05] (Figure 4C). Consistent with the improvement in neurological deficit scores, the mNSS were significantly lower in the EA-treated groups. This finding indicates a notable enhancement in neurological functions, aligning with EA’s proposed benefits in stroke rehabilitation. The funnel plot was made to explore publication bias, and the results showed that there was no publication bias (Supplementary Figure 2H).

## 4 Discussion

This meta-analysis elucidates the considerable neuroprotective potential of EA in the rehabilitation of ischemic stroke, highlighted by a marked reduction in cerebral infarction volume and significant improvements in neurological functions (See Table 2 for details). The substantial decrease in TUNEL-positive cells we observed underscores a notable reduction in apoptosis within ischemic cerebral territories, aligning with contemporary research that explores EA’s impact on apoptotic mechanisms, suggesting a multifaceted neuroprotective strategy encompassing both apoptosis modulation and cerebral perfusion enhancement (11, 26, 30–32).

**Table 2:** A comprehensive overview of the mechanism, with attention to acupoint selection.

Author	year	Acupoints	Mechanisms
Xin-yin Xu	2020	**BL 23** (Shenshu, left, 6 mm depth) **GV 20** (Baihui, 10 mm)	EA exerted its effects by up-regulating the anti-apoptotic protein Bcl-2, down-regulating the pro-apoptotic proteins Bax and caspase-3, and affecting the circadian rhythm proteins Clock and Bmal1. This also suggests that there is a synergy between circadian regulation and neuronal survival pathways in EA’s mechanism of action.
Yu Ri Kim	2013	**GV 20** (Baihui, 0.2 mm-diameter needles were inserted to a depth of approximately 3 mm) **CV 6** (Qihai, 0.2 mm-diameter needles were inserted to a depth of approximately 3 mm)	EA primarily inhibits caspase-3, −8, and −9 activities, which are critical in apoptosis execution, thereby protecting cortical cells from ischemic apoptosis.
Ying Xing	2018	**LI 11** (Quchi, 0.3 mm in diameter with 2–3 mm deep) **ST 36** (Zusanli, 0.3 mm in diameter with 2–3 mm deep)	The intervention of EA is related to the up-regulation of midkine (MK) and the mediation of ERK/JNK/p38 signaling pathway. EA treatment can reduce nerve function deficit, infarct size and neuronal apoptosis.
Heng Zhou	2017	**GV** 20 (Baihui, other details not specified)	EA was found to down-regulate miR-191a-5p, which, in turn, targeted neuronal calcium sensor 1 (NCS-1), enhancing cell viability, reducing apoptosis, and improving neurological outcomes in rats post-stroke.
Siqiao Cao	2021	**GV 26** (Shuigou, with sterile acupuncture needle, 0.25 × 25 mm, and the needle was inserted 0.2 × 0.3 cm.)	The neuroprotective mechanism of EA works through a complex interplay of molecular pathways such as miRNA-34, Wnt signaling, and autophagy processes.
Ranran Ma	2016	**GV 20** (Baihui, other details not specified) **LI 4** (Hegu) **LR 3** (Taichong)	EA can up-regulate anti-apoptotic protein Bcl-2 and down-regulate pro-apoptotic protein Bax to promote cell proliferation. In addition, EA alters the expression balance of MMP-9/TIMP-1, thereby exerting therapeutic effects.
Xiehua Xue	2014	**ST 36** (Zusanli, 0.3 mm in diameter with 2–3 mm deep) **LI 11** (Quchi, 0.3 mm in diameter with 2–3 mm deep)	EA inhibits neuronal apoptosis by activating PI3K/Akt signaling pathway, and can improve nerve function, reduce the volume of cerebral infarction and the number of apoptotic cells, thus playing a neuroprotective role in cerebral ischemia–reperfusion injury.
Bin Chen	2021	**GV 20** (Baihui, other details not specified) **GV 24** (Shenting)	EA can reduce the formation of cofilin rod and the degradation of MAP2, inhibit the cleavage of mitochondrial cofilin and caspase-3, significantly reduce the apoptosis and loss of neurons after ischemic stroke, thus enhancing the recovery of nerve function and reducing brain injury and infarct volume.
Laiting Chi	2018	**GV 20** (Baihui, other details not specified) **GV 14** (Dazhui)	EA can enhance cerebral perfusion, reduce infarct volume, and reduce apoptosis, inflammation, and oxidative stress.
Ruisi Tian	2018	**GV 20** (Baihui, other details not specified)	EA significantly inhibited apoptosis of hippocampal neurons in mice with cerebral infarction by activating Notch3 signaling pathway. This activation leads to increased expression of Notch3 and downstream HES1 genes, thereby helping to inhibit neuronal apoptosis.
Ying Xing	2018	**LI 11** (Quchi, 0.3 mm in diameter with 2–3 mm deep) **ST 36** (Zusanli, 0.3 mm in diameter with 2–3 mm deep)	EA significantly decreased cerebral infarct volume, improved neurological deficits, and reduced apoptotic cell proportions. The treatment modulated the PTEN pathway by up-regulating phosphorylation levels of PDK1, Akt (Thr308), GSK-3β, and down-regulating phosphorylation levels of PTEN, Akt (Ser473).

The bold text in this table indicates acupuncture points. Bold is used for emphasis.

### 4.1 Neuroprotective mechanisms of EA

Current literature suggests that EA’s therapeutic efficacy in cerebral ischemia is driven by complex, multifactorial mechanisms (33). Our synthesis of data from 302 subjects across 11 studies highlights EA’s significant neuroprotective influence, particularly evidenced by the pronounced reduction in cerebral infarction volume [MD = −15.78, 95%CI (−21.40, −10.16), *p* < 0.05]. This observation resonates with Kim et al.’s findings, suggesting that EA’s protective effects might involve local blood flow modulation and neuronal metabolic regulation (22). Moreover, the enhancements in neurological function echo Chi's et al. (13) findings, attributing such improvements to EA’s stimulation of the parasympathetic nervous system, further underscoring the diverse mechanisms of EA’s action. Additionally, the modulation of the MMP-9/TIMP-1 expression balance by EA, crucial for maintaining extracellular matrix integrity and preventing excessive proteolytic activity, suggests another dimension of EA’s neuroprotective mechanism (23, 34).

The notable reduction in TUNEL positive cells observed in our analysis suggests a substantial attenuation of cellular apoptosis within ischemic cerebral regions. This is consistent with the molecular insights provided by Kim et al. (22) and Xing et al. (25, 26), where EA was shown to influence apoptotic pathways, underscoring its potential in curtailing neuronal loss post-stroke. EA’s mechanistic underpinnings, embracing the modulation of apoptosis-centric pathways alongside cerebral hemodynamic enhancements, have been the focal point of preceding inquiries (35–39). But this is not consistent with the results of our analysis. Investigations delineate EA’s modulatory effect over the PTEN signaling cascade, the PI3K/Akt pathway, and the miRNA-34/Wnt/Autophagy axis, implicating these in the apoptotic and autophagic processes within ischemic cerebral detriment (21, 25, 28). Furthermore, EA’s engagement with cofilin—a pivotal apoptotic mediator—through mitochondrial translocation, attenuates ischemic cerebral injury, modulating death receptor (DR) 5 expression and upregulating the inhibitor of apoptosis (IAP) family members cIAP-1 and -2 (15, 22).

The reduction in TUNEL-positive cells, alongside the increase in Bcl-2 relative densities observed in our analysis, suggests a shift toward anti-apoptotic signaling, further evidenced by the significant decrease in the apoptotic index (22, 26, 27, 40). This is in line with literature demonstrating EA’s modulation of key cell survival and apoptosis regulatory pathways, such as the PTEN/PI3K/Akt pathway (21, 28).

EA’s broad-spectrum neuroprotection can be attributed to multifaceted mechanisms, including apoptosis pathway regulation and cerebral perfusion enhancement. The reduction in apoptosis, particularly through the downregulation of caspase-3 and upregulation of Bcl-2, suggests a pivotal role of anti-apoptotic signaling in EA’s neuroprotective effects. It’s noteworthy, however, that our findings regarding caspase-3 relative densities did not exhibit significant differences between the EA and sham-EA groups, hinting at the possibility that EA’s neuroprotective mechanisms may transcend the caspase-3 mediated apoptotic pathways, potentially involving alternative or complementary pathways like those involving Bcl-2, as our analysis indicated a significant increase in Bcl-2 relative densities in the EA treatment group. This is consistent with the findings of Kim et al. (22) and Ma et al. (23), which reported similar modulations in apoptotic regulators following EA treatment. However, the absence of significant differences in caspase-3 densities and Bax levels indicates that EA’s neuroprotective mechanisms extend beyond simple modulation of apoptotic pathways, warranting further investigation into alternative pathways and mechanisms, such as neuroinflammation, autophagy, and neurogenesis (23, 25).

The notable improvements in neurological deficit scores post-EA treatment point toward its efficacy in facilitating neural recovery and functional restoration, consistent with hypotheses suggesting EA’s role in neurorehabilitation via neuroinflammatory response modulation, neurogenesis, and angiogenesis (41, 42). The enhancements in motor, sensory, reflex, and balance functions, as indicated by the significant reduction in mNSS scores, align with Xue's et al. (28) findings on EA’s potential to upregulate neurotrophic factors and enhance synaptic plasticity. These findings suggest EA’s integral role in comprehensive stroke rehabilitation strategies aimed at restoring neurological functions and improving stroke survivors’ quality of life. The improvement in neurological deficit scores and mNSS further accentuates EA’s potential in enhancing functional recovery, a finding that echoes the work of Xu et al. (27), where EA facilitated neural repair and functional rehabilitation through mechanisms that might involve neurogenesis and synaptic plasticity enhancement.

In this study, we included a relatively small number of studies in strict adherence to the inclusion exclusion criteria, and the strict inclusion and exclusion criteria were developed to ensure that the synthesized evidence was of the highest relevance and quality, consistent with the specific objectives of our review. By adhering to these criteria, we aimed to minimize bias and increase the reliability of study results. The limited number of studies may also indicate a lack of high-quality research in this particular area. This suggests the need for more rigorous, well-designed trials to examine the role of electroacupuncture in preventing ischemic stroke.

### 4.2 Innovation

In this study, we systematically integrated the animal experimental data to provide a reference for future clinical study design. In addition, the effect size of electroacupuncture treatment was assessed by quantitative analysis to clarify the influence of different experimental conditions on the effect. Moreover, identifying the potential mechanisms of electroacupuncture effects may provide some basis for advancing basic research and clinical translation.

### 4.3 Clinical implications and future directions

The clinical implications of these findings are profound, suggesting that EA could serve as a viable non-pharmacological intervention in ischemic stroke rehabilitation. The potential for EA to improve functional recovery and reduce neuronal loss opens new avenues for integrative approaches to stroke treatment (43–45). However, these findings hold profound clinical implications, positioning EA as a promising non-pharmacological intervention in ischemic stroke rehabilitation. However, translating these animal model findings to clinical practice necessitates careful consideration of EA protocols, including electroacupuncture points selection, frequency, duration of treatment, and patient-specific factors to optimize therapeutic outcomes.

### 4.4 Limitations and considerations

While this analysis provides robust evidence of EA’s neuroprotective effects, it is not devoid of limitations. The inherent variability in experimental designs and outcome measures across the included studies calls for standardized methodological approaches in future research. Furthermore, addressing the heterogeneity among studies and the predominance of male rodent models will be crucial in refining the evidence base for EA’s role in stroke recovery. Future research should aim to elucidate the molecular and cellular mechanisms underlying EA’s neuroprotective effects further, exploring the potential synergies between EA and conventional stroke therapies.

## 5 Conclusion

This systematic review and meta-analysis highlight the significant neuroprotective effects of electroacupuncture in ischemic stroke models, suggesting its potential as a therapeutic intervention. The primary findings, including the reduction in cerebral infarction volume and apoptosis, coupled with improvements in neurological function, underscore EA’s multifaceted neuroprotective mechanisms. Future research should aim to further elucidate these mechanisms and explore the synergistic effects of EA with conventional stroke therapies, paving the way for innovative integrative treatment strategies in stroke rehabilitation.

## Data Availability

The original contributions presented in the study are included in the article/Supplementary material, further inquiries can be directed to the corresponding author.
