# Supplementary material for: Experimental evidence-based construction of electroacupuncture for ischemic stroke: a meta-analysis and systematic review
Source: Front Neurol. 2025 Feb 5;16:1491132. doi: 10.3389/fneur.2025.1491132 (PMC11835673; doi:10.3389/fneur.2025.1491132)
Supplement: Supplementary file 1 [file Supplementary_file_1.docx]

**Experimental evidence-based construction of electroacupuncture for ischemic stroke: a meta-analysis and systematic review**


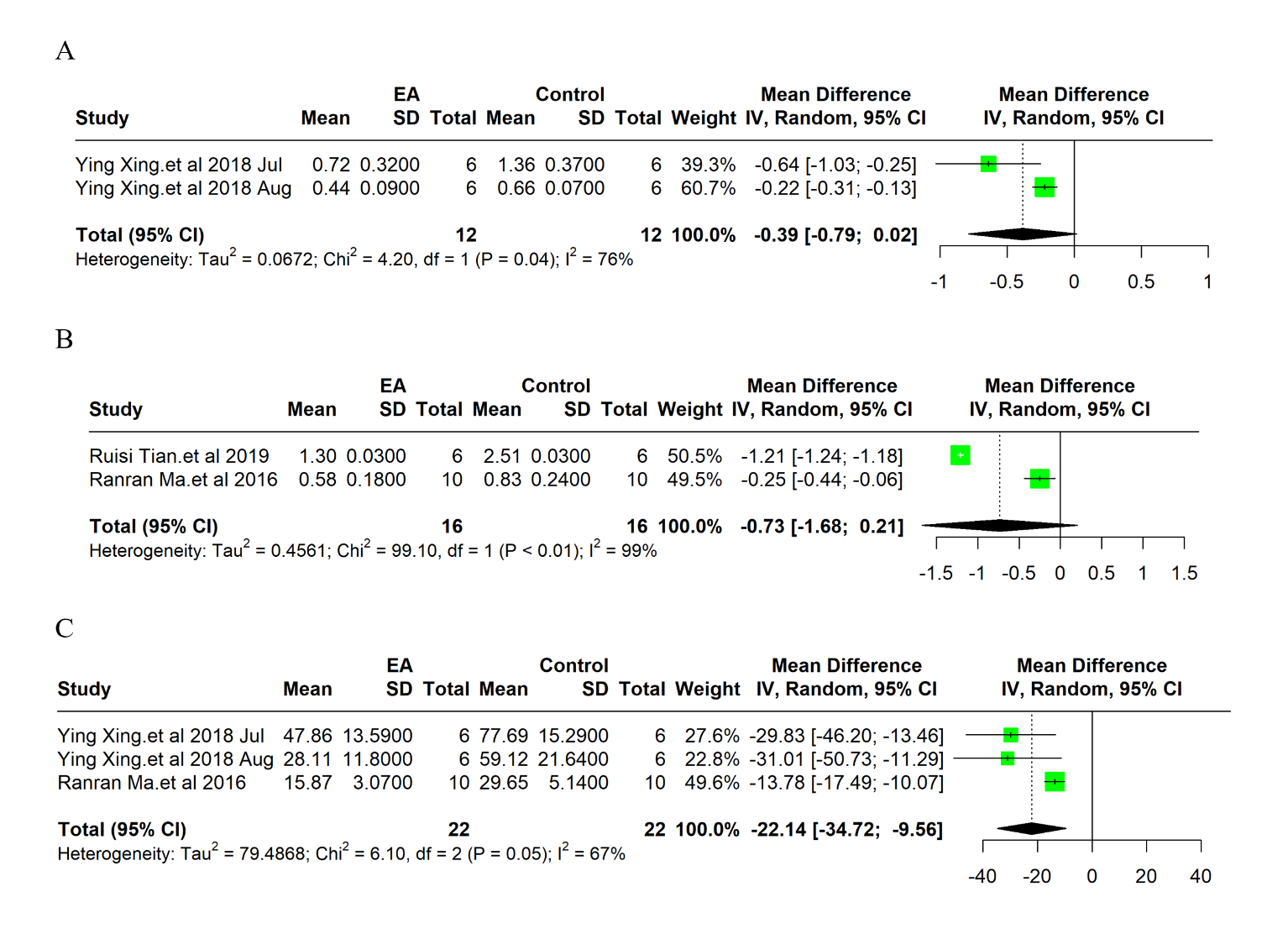
**Supplementary materials**

**Supplementary figure 1.** Meta-Analysis of Electroacupuncture's Efficacy in Ischemic Stroke Models: Forest Plots of Apoptosis indicators. **A)** Forest Plot of Caspase-3 relative densities. **B)** Forest Plot of Bax protein Expression level. **C)** Forest Plot of Apoptotic index

**Supplementary figure 2.** Meta-analysis of the efficacy of electrical therapy in ischemic stroke models: funnel plot. A) Cerebral infarction volume. B)
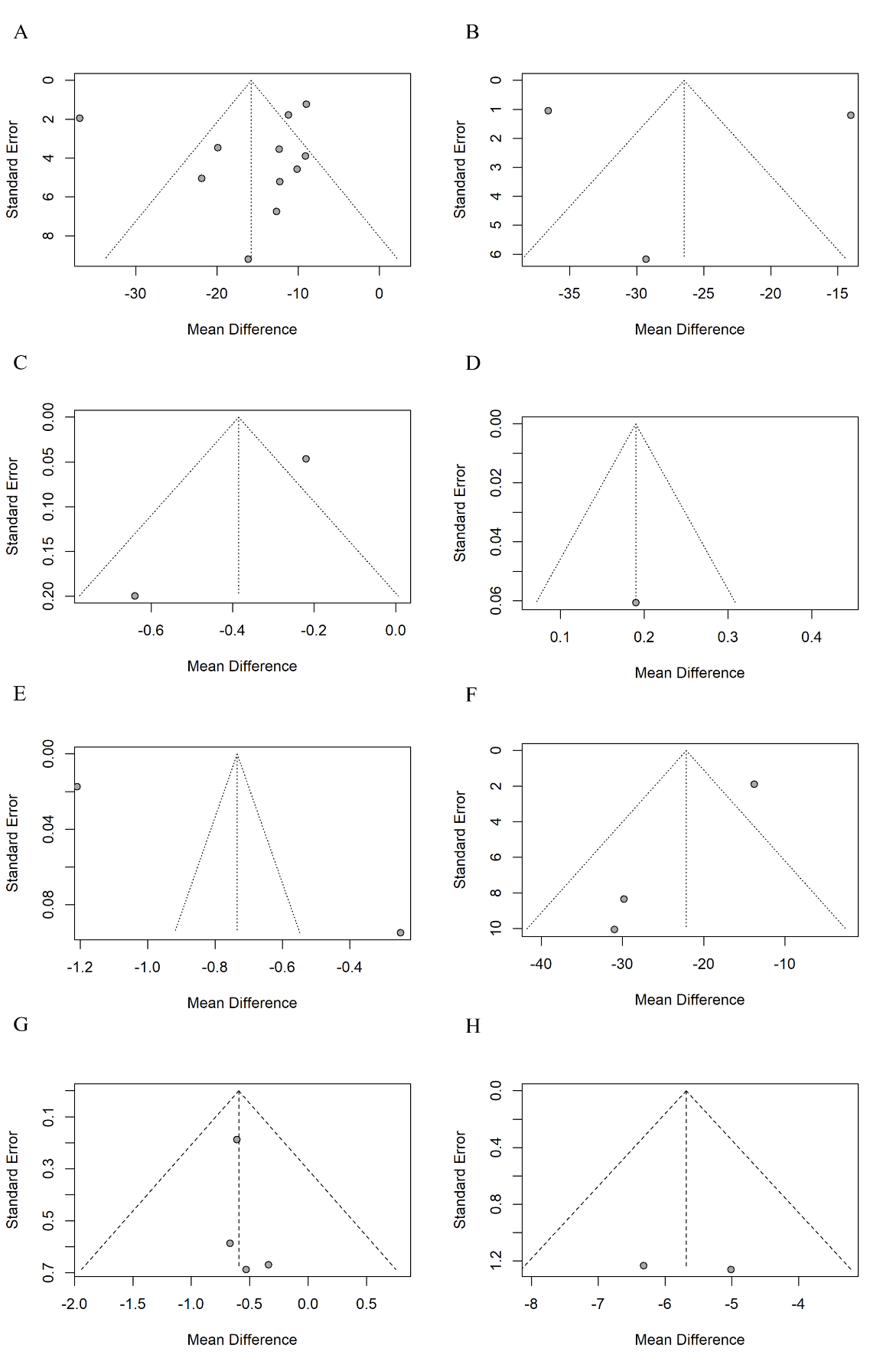
 TUNEL positive cells. C) Caspase-3 relative densities. D) Bcl-2 relative densities. E) Bax protein Expression level. F) Apoptotic index. G) Neurological deficit score. H) Neurological function score of Mnss

**Supplementary figure 3.** Sensitivity analysis of cerebral infarct volume

| **Supplementary table. Abbreviations** | |
| --- | --- |
| ***Abbreviation*** | ***Full Name*** |
| Qu | Quercetin |
| SMD | Standardized Mean Difference |
| 95%CI | 95% Confidence Interval |
| EA | Electroacupuncture |
| TUNEL | The Terminal deoxynucleotidyl transferase (TdT) dUTP Nick-End Labeling |
| mNSS | modified Neurological Severity Scores |
| TTC | Triphenyl tetrazolium chloride |
| MCAO | middle cerebral artery occlusion |
| H&E | Hematoxylin-eosin |
| CAMARADES | Collaborative Approach to Meta-Analysis and Review of Animal Data from Experimental Studies |
| α7nAChR | Alpha-7 nicotinic acetylcholine receptor |
| BrdU | Bromodeoxyuracil |
| GFAP | Glial Fibrillary Acidic Protein |
| Bcl | B-cell lymphoma |
| MK | Midkine |
| ERK | extracellular regulated protein kinases |
| JNK | Jun N-terminal kinase |
| miR | microRNA |
| NCS | Neuronal calcium sensor |
| Ranran Ma | Wingless/Integrated |
| MMP-9 | Matrix metalloproteinase-9 |
| TIMP-1 | Tissue inhibitor of metalloproteinase-1 |
| PI3K | phosphatidylinositol 3-kinase, |
| Akt | Protein kinase B |
| MAP2 | Microtubule-associated protein 2 |
| PNS | Panax notoginseng saponins |
| PTEN | Phosphatase and tensin homolog deletedon chromosome ten |
| GSK-3β | Glycogen synthase kinase-3β |
| PDK1 | Pyruvate Dehydrogenase Kinase 1 |
| DR | Death receptor |
| Bax protein | Bcl-2-associated X protein |
| IAP | Inhibitor of apoptosis |
| cIAP | Cellular inhibitor of apoptosis protein |
| P | Population |
| I | Intervention |
| C | control |
| O | Outcome |
| N | number |
| SCI | Science Citation Index |
| I^2^ | I-square |
| NM | Not mentioned |
